# High energy flexible supercapacitors formed via bottom-up infilling of gel electrolytes into thick porous electrodes

**DOI:** 10.1038/s41467-018-04937-8

**Published:** 2018-07-03

**Authors:** Xiangming Li, Jinyou Shao, Sung-Kon Kim, Chaochao Yao, Junjie Wang, Yu-Run Miao, Qiye Zheng, Pengcheng Sun, Runyu Zhang, Paul V. Braun

**Affiliations:** 1 Micro-/Nano-technology Research Center, State Key Laboratory for Manufacturing Systems Engineering, Xi’an Jiaotong University, 710049 Xi’an, Shaanxi China; 2 Department of Materials Science and Engineering, Frederick Seitz Materials Research Laboratory, and Beckman Institute for Advanced Science and Technology, University of Illinois at Urbana-Champaign, Urbana, IL 61801 USA; 3 School of Chemical Engineering, Chonbuk National University, 567 Baekje-daero, Deokjin-gu, Jeonju-si, Jeollabuk-do 54896 Republic of Korea; 4 Department of Chemistry, University of Illinois at Urbana–Champaign, Urbana, IL 61802 USA

## Abstract

Formation of thick, high energy density, flexible solid supercapacitors is challenging because of difficulties infilling gel electrolytes into porous electrodes. Incomplete infilling results in a low capacitance and poor mechanical properties. Here we report a bottom-up infilling method to overcome these challenges. Electrodes up to 500 μm thick, formed from multi-walled carbon nanotubes and a composite of poly(3,4-ethylenedioxythiophene), polystyrene sulfonate and multi-walled carbon nanotubes are successfully infilled with a polyvinyl alcohol/phosphoric acid gel electrolyte. The exceptional mechanical properties of the multi-walled carbon nanotube-based electrode enable it to be rolled into a radius of curvature as small as 0.5 mm without cracking and retain 95% of its initial capacitance after 5000 bending cycles. The areal capacitance of our 500 μm thick poly(3,4-ethylenedioxythiophene), polystyrene sulfonate, multi-walled carbon nanotube-based flexible solid supercapacitor is 2662 mF cm^–2^ at 2 mV s^–1^, at least five times greater than current flexible supercapacitors.

## Abstract (web summary)

The development of high performance flexible solid supercapacitors calls for an effective approach to infill gel electrolytes into porous electrodes. Here the authors report a bottom-up method to address this technical challenge, which leads to enhanced areal capacitance and durability.

## Introduction

Flexible solid supercapacitors (FSSCs) are usually constructed by sandwiching a gel electrolyte between a pair of porous electrodes. During this process, the gel electrolyte partially infills the pores of the electrodes forming the electrode–electrolyte interface^1–3^. Such designs have drawn attention because of their mechanical flexibility, relatively light weight, potential for low cost, and minimal issues with electrolyte leakage, with the target application typically flexible electronics^1–7^. The FSSC community has primarily focused on advancing the electrode and electrolyte materials in the past decades^8–18^, with only minimal attention paid to developing effective strategies for maximizing formation of the electrode–electrolyte interface (the part of the structure that stores energy). Formation of this interface is the precondition for a high energy density, but remains a considerable obstacle for FSSCs. Current state-of-the-art FSSCs utilize thin electrodes as thin electrodes provide mechanical flexibility and because the required solid electrolyte can infill a thin porous electrode to form a good high area electrode–electrolyte interface. However, thin electrodes, typically ranging from 10s of nm to a few 10s of μm in thickness, tend to have low areal capacitances since capacitance scales with electrode volume^8–23^. For example, while a 500 nm thick electrode formed from a multi-layer graphene/carbon nanotube structure exhibited a volumetric capacitance of 49.5 F cm^–3^, the areal capacitance was only 2.54 mF cm^–2^ ^14^. A 100 nm thick nano-porous gold/polypyrrole electrode exhibited a volumetric capacitance of 150 F cm^–3^, yet its areal capacitance was only 1.8 mF cm^–2^ ^11^. An ultrathin (25 nm thick) graphene electrode had a gravimetric capacitance of 285 F g^–1^, yet the areal capacitance was just 0.45 mF cm^–2^ ^12^.

The obvious solution to increase the areal capacitance is to thicken the electrodes^23^. However, the areal capacitance of reported FSSCs did not scale with electrode thickness, usually saturating while the electrode was quite thin. For example, the areal capacitance of a FSSC based on single wall carbon nanotubes saturates at an electrode thickness of 2~3 μm, probably because the gel electrolyte could only infill the electrode up to this thickness^19,24^. Prior to the report here, the only route to improve the infilling of the electrode was to add macropores to the structure. Examples of FSSC containing both micropores and larger macropores include one formed using 120 μm thick 3D graphene hydrogel electrodes which exhibited an areal capacitance of 372 mF cm^–2^ ^24^, one formed with 81.6 μm thick reduced graphene oxide/polypyrrole nanotube paper electrodes which exhibited an areal capacitance of 512 mF cm^–2^ ^25^, and one formed using graphene/carbon nanofiber aerogel electrodes which showed an areal capacitance of 158 mF cm^–2^ ^26^. In these systems, the macropores both serve as an electrolyte reservoir and improve the infilling of the porous electrodes^24–28^. However, the macropores decreased the overall electrode surface area, decreasing the areal capacitance. To maximize capacitance, an electrode should be thick (e.g., 500 μm), have a minimum of macropores, and maximum of micropores or mesopores. However, prior to the work here, it has not been possible to infill such a structure with solid electrolyte.

Thick pseudocapacitive electrodes based on 3D-structured transition metal oxides provide much higher areal capacitances^29–31^. The areal capacitance of a MnO_2_-coated ~270 μm thick 3D silicon scaffold reached 670 mF cm^–2^ at 2 mV s^–1^ using a 0.5 M Na_2_SO_4_ aqueous electrolyte^29^, the areal capacitance of a microsupercapacitor formed using a ~15 μm thick 3D laser-scribed graphene-MnO_2_ electrode reached 852 mF cm^–2^ using a 1 M Na_2_SO_4_ aqueous electrolyte^30^, and the areal capacitance for 3D-structured 25 μm thick Au/RuO_2_·*x*H_2_O reached 3473 mF cm^–2^ at 0.1 mV s^–1^ in a 0.5 M H_2_SO_4_ electrolyte^31^. While these high areal capacitances would be highly desirable for FSSCs, the brittle nature of metal oxides, coupled with the fact that liquid electrolytes are commonly used for pseudocapacitors, makes building a FSSC using these electrodes challenging.

Starting from our understanding of why it is difficult to infill thick porous electrodes we create a new bottom-up method to infill gel electrolytes into porous electrodes as thick as 500 μm. Using this method, high capacity thick electrodes are infilled with gel electrolytes, and FSSCs are formed from these electrodes. The highest capacity FSSC is realized using bottom-up infilled composite electrodes containing a poly(3,4-ethylenedioxythiophene), polystyrene sulfonate (PEDOT/PSS) blend and multi-walled carbon nanotubes (MWCNT). The FSSC formed using this electrode exhibits an areal capacitance of 2662 mF cm^–2^, about five times greater than current state-of-the-art FSSCs.

## Results

### Top-down vs. bottom-up infilling method

**Fig. 1:**
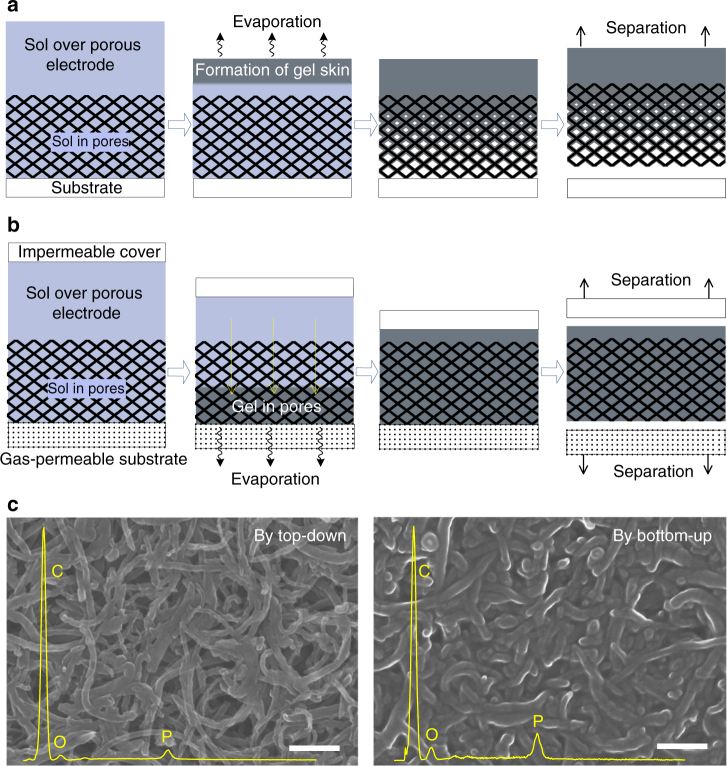
Top-down vs bottom-up infilling. **a** The top-down infilling method. Left to right: sol is casted onto a porous electrode; sol above the porous electrode gels forming a skin; sol in the porous electrode gels, leaving behind voids; a free-standing incompletely filled electrode is obtained after the separation from the substrate. **b** The bottom-up infilling method. Left to right: sol is cast on a porous electrode located on a gas-permeable substrate and covered with an impermeable film; the gel forms from the bottom-up until the entire porous electrode is infilled with gel; a free-standing gel-filled electrode is obtained after removal of the substrate. **c** SEM images of the top of the MWCNT electrodes infilled with gel electrolyte by the top-down (left) and bottom-up (right) methods. Overlaid on the images are energy dispersive spectroscopy scans where the phosphorus peaks indicate the presence of the electrolyte. Scale bar for **c** is 200 nm

Figure 1 illustrates why it is difficult to infill a gel electrolyte into thick porous electrodes, and our suggested solution. As illustrated in Fig. 1a and Supplementary Figure 1, during the top-down gelation process the volume shrinkage, and the dramatic increase in viscosity as the gel point is approached, leads to incomplete infilling. As water evaporates from the electrolyte (a mixture of water, poly(vinyl alcohol) (PVA) and H_3_PO_4_), a gel layer is generated at the air-electrolyte interface (the first two snapshots in Supplementary Figure 1), which thickens as the water continues to evaporate. As shown by the last two snapshots in Supplementary Figure 1, the gel is sufficiently solid that it can be mechanically removed from the top of the electrolyte solution. Because the gel forms from the top-down, it is very difficult to infill a thick porous electrode via the commonly used top-down infilling process (i.e., via drop casting) (Fig. 1a). As the water evaporates, a viscous skin forms above the electrode as illustrated in Fig. 1a. Because the volume of PVA and H_3_PO_4_ in the liquid below the skin starts out as typically <10%^32^, once this liquid dries in the gel, only a small proportion of the electrode pore volume is occupied by the gel and most of the pore space is empty. As the pore sizes in the electrode become smaller, the infilling problem only becomes worse, because viscous resistance to flow grows^33,34^.

In the bottom-up infilling method, the electrolyte solution is directly cast onto a porous electrode located on a gas-permeable substrate (Fig. 1b). A water impermeable film (e.g., polyester (PET)) is then placed over the electrolyte solution, forcing the water in electrolyte solution to evaporate downwards through the gas-permeable substrate. The downward evaporation of water starts the formation of the gel electrolyte from the bottom of the porous electrode; the gel then grows upwards into the electrode. No voids form in the pores of the electrode because capillary forces drive the liquid solution to continuously refill the pores, as indicated by the yellow arrows in the second scheme in Fig. 1b. Since the capillary force is inversely proportional to the pore size, as an added benefit of this infilling approach, we suspect smaller pore electrodes may infill better than larger pore electrodes. After removing the impermeable cover and the gas-permeable substrate, a flexible electrode with a well-infilled gel electrolyte is obtained.

### Electrode infilling

Because of their high surface area, stability, mechanical flexibility, and electrical conductivity, carbon nanotube-based electrodes are commonly used for FSSC^2,4,35–37^. However, these electrodes usually have small and complex pores which are difficult to infill with a gel electrolyte. For the study here, we specifically use a thick (typically ~500 μm) electrode formed from randomly distributed multi-walled carbon nanotubes (MWCNTs, ~50 nm in diameter). Figure 1c shows scanning electron micrograph (SEM) images of MWCNT electrodes infilled with gel electrolyte by the top-down and bottom-up methods. Using the bottom-up infilling method, the gel electrolyte infills the entire MWCNT electrode and minimal voiding is observed. Using the top-down infilling method, the amount of gel electrolyte in the porous electrode decreases significantly from the bottom to the top of the electrode (Supplementary Figure 2). Phosphorus mapping via energy dispersive spectroscopy (EDS) also suggests a different degree of gel infilling (the gel contains H_3_PO_4_); compare the intensity of the P peak in the spectrums in Fig. 1c. Using the bottom-up method, the atomic ratio between carbon and phosphorus is nearly constant at 1:0.051 across the cross section of the MWCNT electrode, indicating a uniform distribution of gel electrolyte. Using the top-down infilling method, the value decreases from 1:0.05 at the bottom of the electrode to 1:0.014 at the top of the electrode, suggesting a significant decline of gel infilling from bottom to the top of the porous electrodes. The degree of gel infilling of the electrode pores can be quantified using Brunauer–Emmett–Teller surface area analysis and Barrett–Joyner–Halenda pore size and volume analysis (Supplementary Figure 3). The pore volume of the electrode infilled using the bottom-up method is half than that infilled using the top-down method, and the pore sizes are also significantly decreased for the bottom-up infilled electrode. The difference in the gel infilling of the electrode by the top-down and bottom-up methods is quantitatively determined via a simple test using a micrometer (Supplementary Figure 4).

### FSSC electrical properties

**Fig. 2:**
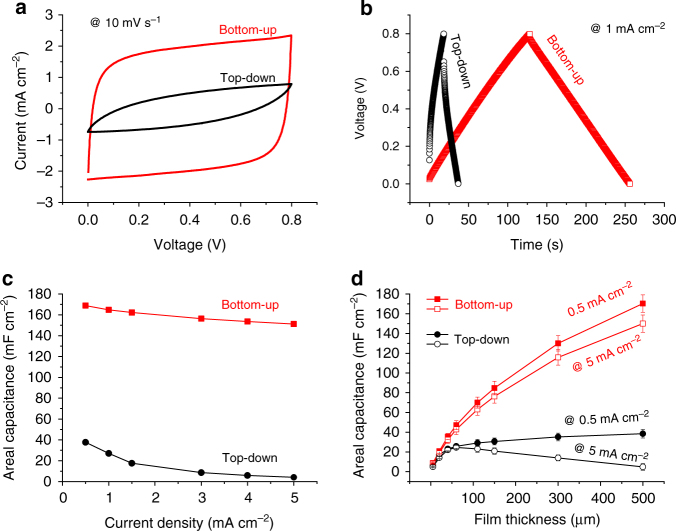
Electrochemical properties of the top-down and bottom-up infilled FSSCs. **a** CV and **b** GCD curves of the different FSSCs. **c** Areal capacitances of the top-down and bottom-up infilled FSSCs at different current densities. **d** Thickness dependence of the areal capacitance for top-down and bottom-up infilled FSSC devices at 0.5 and 5 mA cm^–2^

Figure 2 shows the electrical properties of FSSCs formed via top-down and bottom-up infilling of 500-μm-thick MWCNT electrodes. Figure 2a, b and Supplementary Figure 5 show two-electrode cyclic voltammetry (CV) and galvanostatic charge-discharge (GCD) curves for FSSC formed using the two-electrode preparation methods. The FSSC fabricated by the top-down method shows a much smaller capacitance than one formed by the bottom-up method. There are several reasons for this. First, the top-down infilled electrode is incompletely filled, and thus part of the structure is not contributing to the capacitance. Second, in the partially filled regions, the ions have to bypass the voids during charging and discharging, leading to long diffusion paths, and consequently an increased equivalent series resistance (ESR) which tends to result in a lower power and energy capability^38^. The ESR for the bottom-up (9.3 Ω cm^2^) and top-down (96 Ω cm^2^) infilled FSSC can be obtained from the Nyquist plots (Supplementary Figure 6)^39^.

As shown in Fig. 2c, the areal capacitance and rate performance of the bottom-up infilled FSSC is significantly greater than the top-down infilled FSSC. The areal capacitance of the bottom-up infilled FSSC is ca. 164.8 mF cm^–2^ at a current density of 1 mA cm^–2^, which is six times the 27 mF cm^–2^ exhibited by the FSSC formed by top-down infilling at the same current density. Clearly, increasing the degree of infilling of the gel electrolyte in the thick porous electrode increases the accessible surface area of the electrode and thus the overall capacity^40,41^. Along with an improvement in capacity, the rate performance (Fig. 2c) is also significantly improved by the bottom-up infilling method as estimated from the GCD profiles at current densities ranging from 0.5 to 5 mA cm^–2^. The areal capacitance for the bottom-up infilled FSSC is ca. 168.8 mF cm^–2^, at a discharge current 0.5 mA cm^–2^, which is nearly five times the capacitance of the FSSC formed by the top-down infilling method at the same current density. The capacitance of bottom-up infilled FSSC remains as high as 151.2 mF cm^–2^ at the high discharge current of 5 mA cm^–2^ (89% capacity retention relative to the 0.5 mA cm^–2^ discharge). In contrast, the top-down infilled FSSC retains only 10% of its capacitance (4.1 mF cm^–2^) under the same test conditions. This difference in rate ability is also apparent in the frequency-dependent impedance measurements (Supplementary Figure 6).

The bottom-up method enables scaling of the areal capacitance with electrode thickness. Figure 2d shows the dependence of the areal capacitance with thickness as a function of current density and infilling method. For the top-down method, the areal capacitance saturates with increasing electrode thickness at a current density of 0.5 mA cm^–2^, similar to other literature reports^19,24,42^. At the higher current density of 5 mA cm^–2^, the capacitance even starts to decline once the electrode is thicker than about 50 μm (Fig. 2d), probably due to kinetic limitations caused by voids in the electrode. In contrast, using the bottom-up infilling method, the areal capacitance increases with electrode thickness until at least 500 μm. It should be noted that the areal capacitance does not linearly increase with the electrode thickness, in particular at high current density, because the mean ion diffusion length increases with electrode thickness. Since the volume of the MWCNT electrode per square centimeter is directly proportional to the thickness, the volumetric capacitance of FSSCs shows similar trends as the thickness dependence (Supplementary Figure 7). At 10 mA cm^–3^ for example, the volumetric capacitance for the top-down infilled FSSC, on an electrode volume basis decreases from 6.1 F cm^−3^ for the 5 μm thick electrode to 0.39 F cm^−3^ for the 500 μm thick electrode, while, the volumetric capacitance for the bottom-up infilled FSSC decreases significantly less, from 6.37 to 1.71 F cm^−3^. We find the volumetric capacitance of even the thick (>100 μm) bottom-up infilled FSSC is comparable to the value of other previously reported, much thinner, sandwich type MWCNT-based thin-film FSSCs^43–45^.

The electrochemical performances of FSSCs infilled with the gel electrolyte were additionally compared over a voltage window of 0 to 1 V with the same electrode filled with 1 M H_2_SO_4_ aqueous electrolyte (Supplementary Figure 8). As anticipated, the capacitance, rate performance and ESR of the liquid electrolyte filled electrode was better than for the electrodes infilled with the gel electrolyte because of the better ion conductivity of the aqueous electrolyte than the gel electrolyte. However, liquid electrolytes lead to serious packaging challenges for flexible systems.

### Mechanical performance

**Fig. 3:**
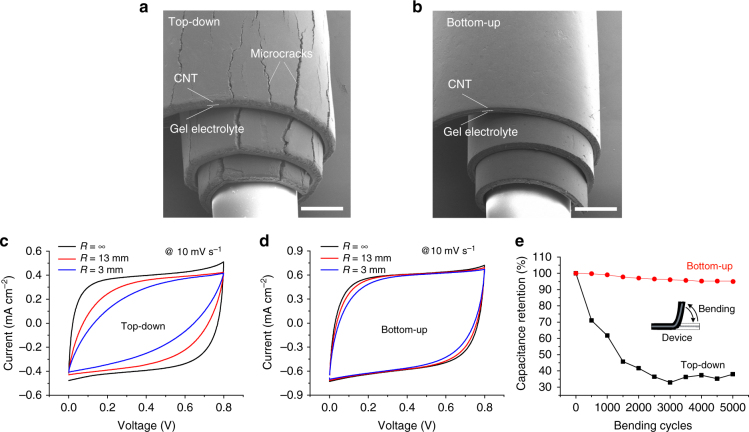
Gel filling method dependent mechanical stability. **a**, **b** The gel infilling-dependent microcracks (or lack of microcracks) in ~150 μm in thick MWCNT electrodes rolled-up over a glass tube with a radius of ~0.5 mm fabricated by the two methods. Electrochemical performance of **c** and **d** once rolled-up FSSC devices and **e** after repeated bending. In **e** the capacitance retention with the bending number is confirmed by GCD at a current density of 0.15 mA after every 500 bends at bending radius of curvature of 5.7 mm. Scale bar for **a** and **b** is 500 μm

For practical application of FSSCs, mechanical performance is important^1–3,9^. We find the improved infilling of gel electrolyte in the porous electrode by the bottom-up method significantly improves mechanical flexibility and stability (Fig. 3). The PVA/H_3_PO_4_ gel polymer electrolyte is flexible and elastic at room temperature, and serves to bind the electrode together^46^, which improves the mechanical properties of the porous electrode. As shown in Fig. 3a, b, the top-down infilled electrodes exhibit microcracks with bending, while the bottom-up infilled electrode exhibits no cracking even the electrode is rolled-up over a glass tube with a radius as small as 0.5 mm. Even if they do not lead to complete failure, microcracks are problematic as they will increase the ESR and may even electrically isolate parts of the electrode, which will harm the device electrochemical performance. Figure 3c, d compare CV curves of rolled-up FSSC devices formed from ~150 μm thick electrodes infilled by the two different methods. For a FSSC device formed by the top-down method, the cathodic/anodic current values decrease with a decreasing rolled-up radius, which we ascribe to electrode cracking. The CV curves also become significantly distorted as the rolled-up radius decreases, indicative of a crack-induced large ESR^47^. The ESR values increased from 28.7 Ω cm^2^ before bending, to 108.8 Ω cm^2^ for a bending radius, *R*, of 13 mm, to 252.6 Ω cm^2^ for *R* = 3 mm, as calculated from the IR drops on GCD plots at the corresponding radii (Supplementary Figure 9). The bottom-up infilled structures exhibit considerable stability to mechanical cycling (Fig. 3e). The capacitance retention of the thick FSSC with the well-infilled gel electrolyte is above 95% after 5000 bending cycles with a radius curvature of 5.7 mm, while the capacitance retention is only 40% for the FSSC with less infilled gel electrolyte. In contrast to the top-down infilled structures, CV curves of the bottom-up infilled rolled-up FSSC remain almost unchanged as a function of rolled-up radii (Fig. 3d), demonstrating the electrode stability under different bending stresses. The difference in capacity of the two-electrode designs is almost certainly due to microcracks in the less infilled electrodes (Fig. 3a and Supplementary Figure 10), which increases the ESR from 28.7 Ω cm^2^ before the first bending cycle to 107 Ω cm^2^ after 5000 bending cycles (Supplementary Figure 11).

### Practical considerations

For practical application, the FSSC electrochemical stability, leakage current and self-discharge rate are important. The thick bottom-up infilled FSSCs show good performance in all these areas. After 10,000 GCD cycles at a constant current of 10 mA cm^−2^, a 150 μm thick bottom-up infilled FSSC retains 98% of initial capacitance and a columbic efficiency near unity, indicating a good cycle life (Supplementary Figure 12). The leakage current was measured to be 2.5 μA after 12 h (Supplementary Figure 13), which is comparable to commercial supercapacitors^40^. Specifically, the time required for the voltage across the supercapacitor to decrease from *V*_max_ (i.e., 1 V) to 1/2*V*_max_ is >24 h (Supplementary Figure 13) compared to 8–21 h for commercial supercapacitors^48^.

### Tandem devices

Tandem bottom-up infilled FSSCs are configured in series and in parallel to increase the voltage window or capacitive current, respectively (Supplementary Figure 14). The tandem serial FSSCs are operated over a 1.6 V window (twice that of a single FSSC), and as expected, there is a reduction in the current because of the increased series resistance, as confirmed by CV and GCD (Supplementary Figure 14). The tandem parallel FSSCs provided twice the current of a single FSSC, while maintaining a steady capacitive behavior throughout the 0.8 V voltage window. After charging at 3 V for 24 min, a device of three FSSCs linked in series can light a blue LED for ~10 min (the LED turn-on voltage is ∼2 V, thus the requirement of several FSSCs in series) (Supplementary Figure 15a). Owing to the mechanical stability of the bottom-up infilled FSSCs, the device of three FSSCs linked in series can be rolled around a cylindrical pencil (radius of ~4 mm) while still powering the LED (Supplementary Figure 15b).

### Bottom-up infilled pseudocapacitor

**Fig. 4:**
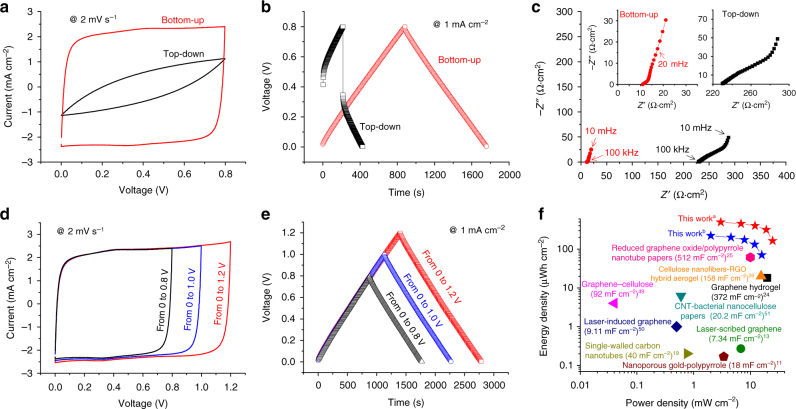
Electrical performance of FSSCs formed using 500 μm thick PEDOT/PSS-WMCNT electrodes. **a** CV, **b** GCD profiles, and **c** Nyquist plots (100 kHz to 10 mHz) of the top-down and bottom-up designs. Note that the inset is the plot for the bottom-up infilled FSSC. **d** CV and **e** GCD curves over different potential windows for a FSSC formed from bottom-up infilled 500 μm thick PEDOT/PSS-MWCNT electrodes. **f** Areal Ragone plot (per cm^2^ of the FSSC device). This work^a^ and this work^b^ refers to the bottom-up infilled FSSCs with an electrode thicknesses of 500 μm tested over potential windows of 0 to 1.2 V and 0 to 0.8 V, respectively

To demonstrate the bottom-up infilling versatility, a composite 70 wt% PEDOT/PSS 30 wt% MWCNT electrode (Supplementary Figure 16), is used for fabricating FSSCs. In this design, the conductive polymer, PEDOT/PSS, enhances the mechanical properties by serving as a binder for the MWCNTs, and the capacitance is increased due to the pseudocapacitive properties of PEDOT. The composite electrode has good mechanical flexibility (Supplementary Figure 17), and perhaps more importantly, a considerable mechanical strength. The yield stress is at least 7 MPa (Supplementary Figure 17), which should make it attractive for FSSCs. The electrochemical performances of FSSCs formed using 500-μm-thick top-down and bottom-up infilled PEDOT/PSS-MWCNT electrodes are compared (Fig. 4). The bottom-up infilled FSSC based on 500-μm-thick PEDOT/PSS-MWCNT electrodes exhibits rectangular-shaped CV curves (Fig. 4a, Supplementary Figure 18a) and symmetric triangular-shaped GCD profiles (Fig. 4b, Supplementary Figure 18b), while the top-down infilled FSSC provides distorted CV curves and asymmetric GCD profiles at the same voltage scan rate and current density. The differences are particularly obvious in the Nyquist plots (Fig. 4c). The ESR for bottom-up infilled FSSC is 11.2 Ω cm^2^, while for the top-down infilled FSSC it is 231.3 Ω cm^2^.

The areal capacitance of the bottom-up infilled FSSC cell formed from the 500 μm thick electrodes is 2662 mF cm^–2^ at 2 mV s^–1^ (Fig. 4a), at least five times greater than the previously reported state-of-the-art (500 mF cm^–2^ or less)^9–19,24–26,49–51^. The 500 μm thick bottom-up infilled FSSC retains an areal capacitance of 2038 mF cm^–2^ at a scan rate of 10 mV s^–1^ (Supplementary Figure 18). In comparison, the areal capacitance of the 500 μm thick top-down infilled FSSC decreased from 582.6 mF cm^–2^ at 2 mV s^–1^ to 55.7 mF cm^–2^ at 10 mV s^–1^ (Supplementary Figure 18). It is also notable how much greater the areal capacitance of the bottom-up infilled PEDOT/PSS-MWCNT-based FSSC is than one formed using only MWCNT (compare Fig. 2a, b with Fig. 4a, b). This difference is perhaps due to the presence of the electrically conductive PEDOT, and probably more importantly the pseudocapacitance of PEDOT^52,53^. The loading of PEDOT/PSS in the composite electrodes influences the capacitance of FSSCs and we found the optimal value to be ~70 wt.% in our experiments (Supplementary Figure 19). The bottom-up infilled PEDOT/PSS-MWCNT-based FSSC also provides a good rate performance relative to a top-down infilled PEDOT/PSS-MWCNT electrode (Supplementary Figure 20a) and provides a capacitance retention of >93% after 10,000 charging/discharging cycles at a current density of 15 mA cm^–2^ (Supplementary Figure 20b).

When the cell voltage increased from 0.8 to 1.2 V, CV curves of FSSC formed using bottom-up infilled 500-μm-thick PEDOT/PSS-MWCNT remained rectangular (Fig. 4d), and their GCD profiles remained symmetric and triangular (Fig. 4e). When the FSSC was charged/discharged between 0 and 1.2 V at 2 mV s^–1^, the areal energy and power densities were 539.4 μWh cm^–2^ and 3.24 mW cm^–2^, respectively. At 50 mV s^–1^, over the potential window of 0 to 1.2 V, the energy density was 165 μWh cm^–2^ and the power density was as high as 24.75 mW cm^–2^. As illustrated on a Ragone plot (Fig. 4f), this performance significantly exceed the current state-of-the-art^9–19,24–26,49–51^. For example, a FSSC formed from cellulose nanofiber–graphene aerogel electrodes provided only 18.1 μWh cm^–2^ at power density of 19.9 mW cm^–2^^26^ and a FSSC formed using 85.6-μm-thick reduced graphene oxide/polypyrrole nanotube paper electrodes provided 61.4 μWh cm^–2^ at power density of 10 mW cm^–2^ ^25^.

## Discussion

A bottom-up infilling method is demonstrated to be highly effective for infilling of solid gel electrolytes into porous electrodes. This approach turns the commonly problematic skin formation and volume shrinkage issue during the gel formation into an advantage rather than a disadvantage. Because the bottom-up infilled gel electrolyte deeply penetrates the porous electrode, the accessibility of ions to the internal electrode surface is maximized and the ion diffusion path length is minimized, resulting in a significantly improved electrochemical performance relative to most other thick FSSCs. Because the polymer gel mechanically interlocks the porous electrode, these thick electrodes are mechanically robust and can be rolled and repeatedly bent without a reduction in capacitance. We believe this bottom-up electrode infilling strategy will be broadly useful for loading gel electrolytes into different porous electrodes because of the near-universality of the volume shrinkage of a gel electrolyte during solvent removal. We note that perhaps areal capacitances even higher than the present best value (2662 mF cm^–2^) may be possible using advanced porous pseudocapacitor electrodes^29–31,54–56^ if they can be formed into thick structures.

## Methods

### Gelation observations

0.4 mL of PVA/H_3_PO_4_ electrolyte solution is dropped onto the hydrophobic silicon wafer at room temperature for the observation of the gelation process. The PVA/H_3_PO_4_ electrolyte solution was prepared as follows. 1 g of PVA (Mw ∼95,000 g mol^−1^, 95% hydrolyzed, Acros) is dissolved in 15 mL of deionized water at 90 °C with vigorous stirring until the solution became transparent. After cooling to room temperature, 0.8 g of H_3_PO_4_ (85 wt.% aqueous solution, Aldrich) is added into the solution and stirred for 12 h at room temperature forming a homogeneous solution. For a better observation of the electrolyte solution droplet, the silicon wafer was treated in a 1 wt% toluene solution of heptadecafluorodecyltrimethoxysilane (FAS) for 1 h to make the surface hydrophobic to ensure a proper height (i.e., a larger contact angle) of the drop of electrolyte solution.

### Fabrication of FSSCs

(1) Fabricating MWCNT electrodes by the bottom-up method: 9%wt. MWCNT solution (9–10 mg mL^–1^, Aladdin) is casted onto a horizontally placed glass substrate and dried at room temperature for 12 h to form a MWCNT film, whose thickness depends on the casted amount of MWCNT solution. Then the MWCNT film on the glass substrate is baked at 150 °C for 2 h to reduce the electrical resistance. Then the MWCNT film is removed from the substrate by a stream of deionized water. Next, the free-standing MWCNT film is placed onto a polyvinylidene fluoride (PVDF) millipore membrane (0.1 μm pore size, Fisher Scientific), which is located on a loose sponge. A sufficient amount (0.8 μL cm^–2^ μm^–1^) of H_3_PO_4_/PVA/H_2_O electrolyte solution to fill the MWCNT film is then cast onto the film. Next, a 60 μm thick PET film is placed on top of the electrolyte solution. After 24 h at room temperature, the sol electrolyte gelled and a free-standing gel electrolyte-infilled MWCNT, and adhered by a layer of gel electrolyte, is obtained by removing the PVDF substrate and the PET cover. The free-standing gel electrolyte infilled MWCNT electrodes are further dried at 60 °C for 12 h.

(2) Fabricating MWCNT electrodes by the top-down method: the MWCNT solution is drop cast onto a glass substrate, dried at room temperature for 12 h, and then baked at 150 °C for 2 h. The same volume of electrolyte solution as for the bottom-up infilled electrode, 0.8 μL cm^–2^ μm^–1^, is drop cast onto the MWCNT electrode. After 24 h at room temperature, the sol electrolyte gels and a free-standing MWCNT, containing the gel electrolyte in the pores and adhered by a layer of gel electrolyte, was obtained after separation from the glass substrate. Then the free-standing MWCNT electrode is further dried at 60 °C for 12 h.

(3) FSSC device assembly: The prepared electrodes are cut into small pieces with areas of 1 cm^2^. A small amount of electrolyte solution is bladed onto the gel surface of the as-prepared MWCNT electrodes, serving as a glue. Another electrode is placed face-to-face onto the electrolyte solution layer. Then a mechanical force (10 kg cm^–2^) is applied to press the electrodes pair firmly together to ensure good contact between the gel layer. Finally, Cu tapes are adhered to the bare MWCNT electrode surfaces, serving as current collectors for the electrochemical testing.

(4) Fabrication of PEDOT/PSS-MWCNT-based FSSCs: A slurry of PEDOT/PSS and MWCNT is prepared as follow: 30 mL PEDOT/PSS aqueous solution (1.3 wt.%, Aldrich) is mixed with MWCNT aqueous solution (9 wt.%, Aldrich) by a volume ratio depending on the different concentration of PEDOT/PSS in the electrodes. The mixtures were vigorous stirred under 60 °C for 3 h, partially evaporating the water in the mixture to form viscous slurry. The viscous slurry is painted onto a PVDF millipore membrane, dried under room temperature for 12 h, and then baked at 120 °C for 20 min to form a PEDOT/PSS-MWCNT electrode. The PEDOT/PSS-MWCNT electrodes are infilled with the gel electrolyte by bottom-up (or top-down) processes and assembled into FSSC cells as described above in ‘FSSC device assembly’ section.

### Characterization and measurements

The infilling of gel electrolyte in the pores of the MWCNT electrodes is investigated by means of field emission scanning electron microscopy (SEM, Hitachi, S-4700), energy dispersive spectroscopy (Hitachi, S-4700) and BET (NOVA 2200e). Electrochemical characterization, including CV, GCD, and EIS, is performed using a VMP3 multichannel potentiostat (VMP3, Bio-Logic, USA) and a potentiostat (Versastat 3, Princeton Applied Research, USA) in the two-electrode mode at room temperature. Contact electrodes, i.e., Cu tapes, are adhered to the FSSC devices for an easy connection to the probe and convenient tandems. The thicknesses of the MWCNT electrodes were measured using the cross-sectional SEM and a micrometer. Calculations of specific capacitance and energy and power densities are discussed in detail in Supplementary Note 1.

### Data availability

Data used in this study is available from the corresponding authors upon request.

## Electronic supplementary material

Supplementary Information
