# Supplementary material for: High energy flexible supercapacitors formed via bottom-up infilling of gel electrolytes into thick porous electrodes
Source: Nat Commun. 2018 Jul 3;9:2578. doi: 10.1038/s41467-018-04937-8 (PMC6030180; doi:10.1038/s41467-018-04937-8)
Supplement: Supplementary file 1 — Supplementary Information [file 41467_2018_4937_MOESM1_ESM.pdf]

Supplementary Information

High energy flexible supercapacitors formed via bottom-up infilling of gel electrolytes into thick porous electrodes

Li et al.

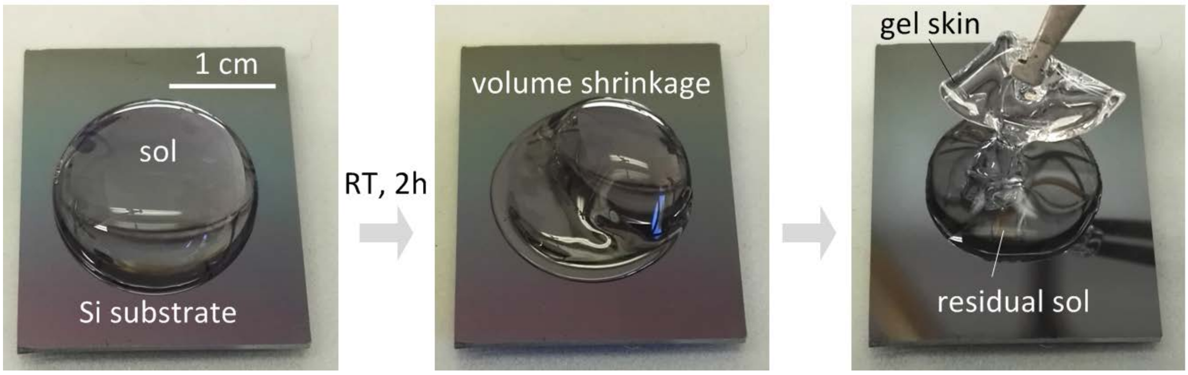

Supplementary Figure 1 | Snapshots as the sol dries forming a gel skin. Left to right: the initial droplet of electrolyte solution (containing water, PVA and H_3PO_4) on the Si substrate; the reduced volume after 2 h at room temperature; removal of the gel formed at the surface.

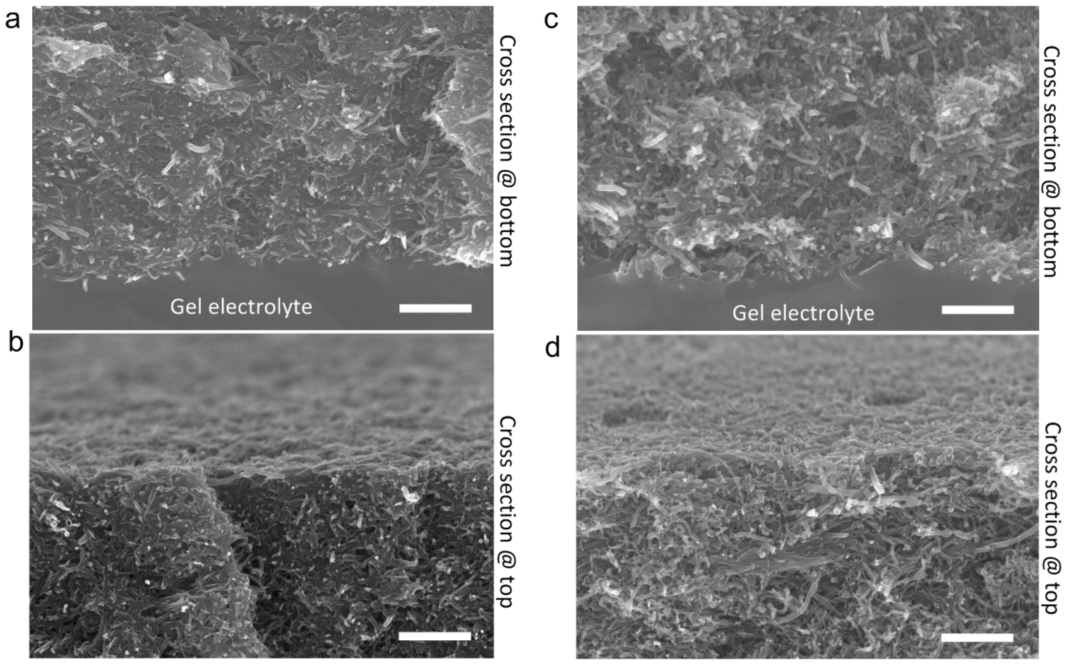

Supplementary Figure 2 | Comparison of gel electrolyte infilling into MWCNT electrodes by the bottom-up and top-down methods. SEM images showing the difference in infilling of the gel electrolyte in the MWCNT electrodes by the bottom-up (a, b) and top-down method (c, d) at different positions of across the 500-μm-thick MWCNT electrodes, as the noted in the images. Scale bars: 1 μm.

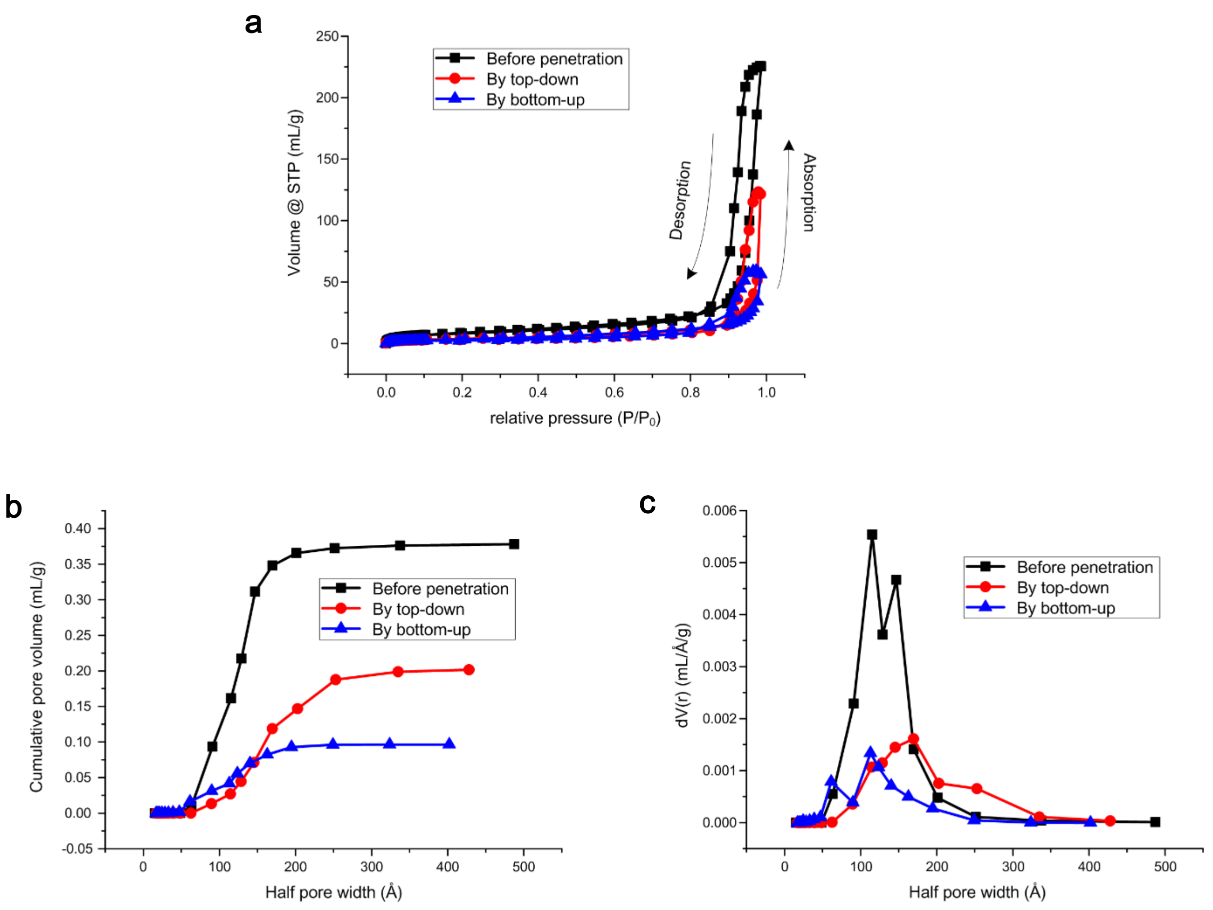

Supplementary Figure 3 | Measurements of the pores of the MWCNT electrodes before and after infilling of gel electrolyte by the top-down and bottom-up methods. (a) The nitrogen adsorption isotherms for MWCNTs electrodes before and after the infilling of gel electrolyte by different methods. The volume of the pores in the top-down MWCNT electrodes was nearly 2 times larger than that in the bottom-up MWCNT electrodes (b), indicating the difference in the infilling of gel electrolyte by the two different methods. The pore sizes of the electrode were also significantly decreased by the bottom-up relative to the top-down method (c).

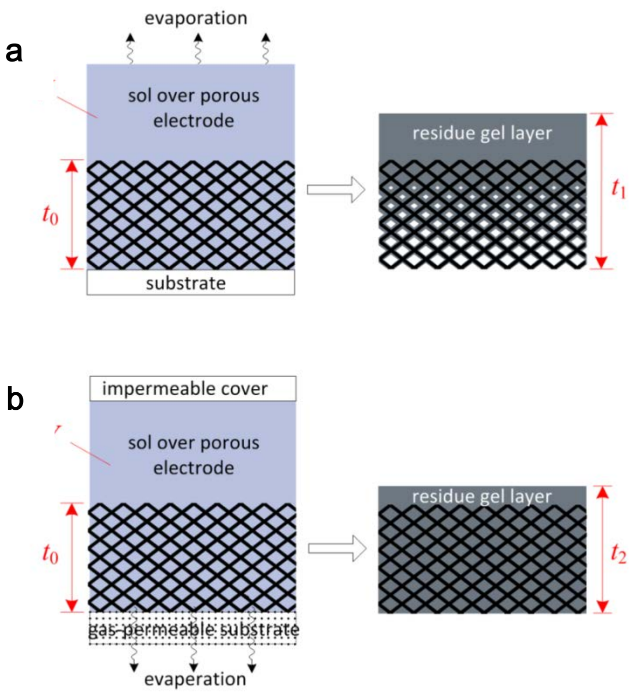

Supplementary Figure 4 | Quantitative evaluation of the degree of infilling by the top-down and bottom-up methods. When the same amount of sol solution of (V) is cast onto MWCNT electrodes with the same thickness (t_0) and area (S) using the two different infilling processes, different thicknesses ($t_1 < t_2$) of the MWCNT electrodes together with residue gel layer can be determined by simply using a micrometer. Note t_1 for top-down method (a) and t_2 for the bottom-up method (b). For example, when t_0 = 500 µm, V = 8.8 mL, S = 22.2 cm^2, it was measured that t_1 = 678 ± 10 µm and t_2 = 638 ± 10 µm. This result indicates that the residual gel layer formed by the bottom-up method was about 40 µm thinner than that formed by the top-down method because more gel was filled into the MWCNT structured by the bottom-up method than that by top-down method.

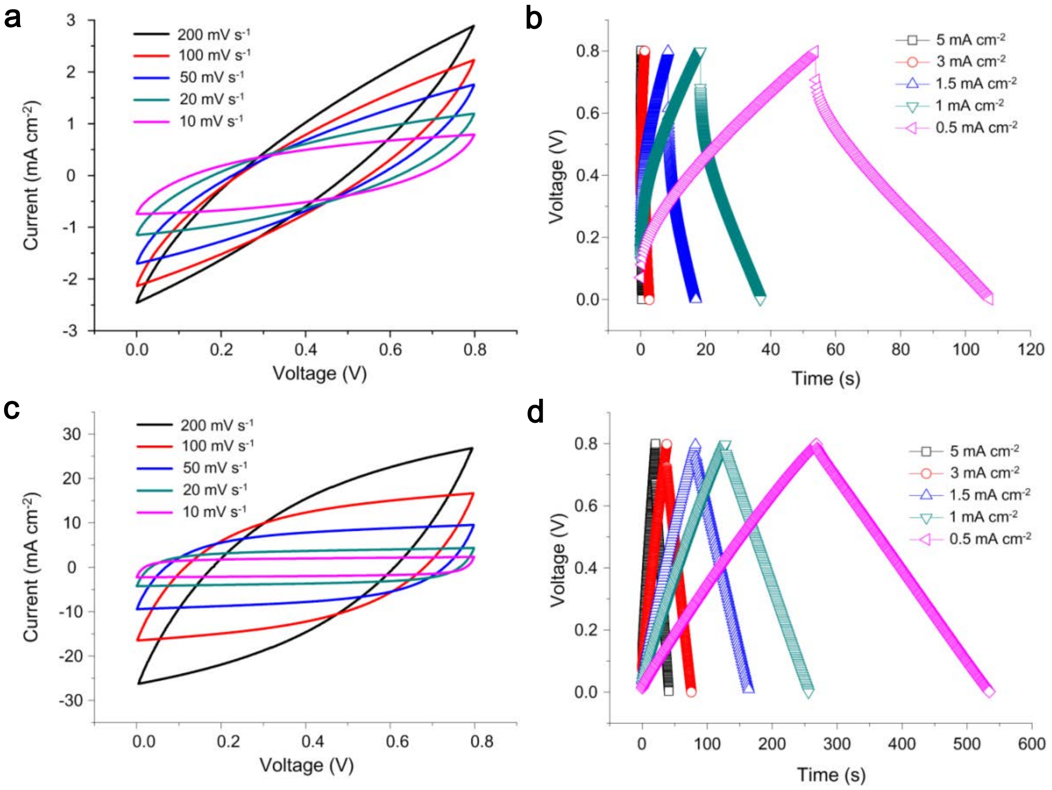

Supplementary Figure 5 | Comparison of electrochemical performance of FSSCs formed by different infilling methods. The CV and GCD curves of FSSCs by top-down (a, b) and bottom-up (c, d) infilling methods. The thickness of each electrode in the FSSCs was ~ 500 μm and the area of each FSSC was ~ 1 cm^2.

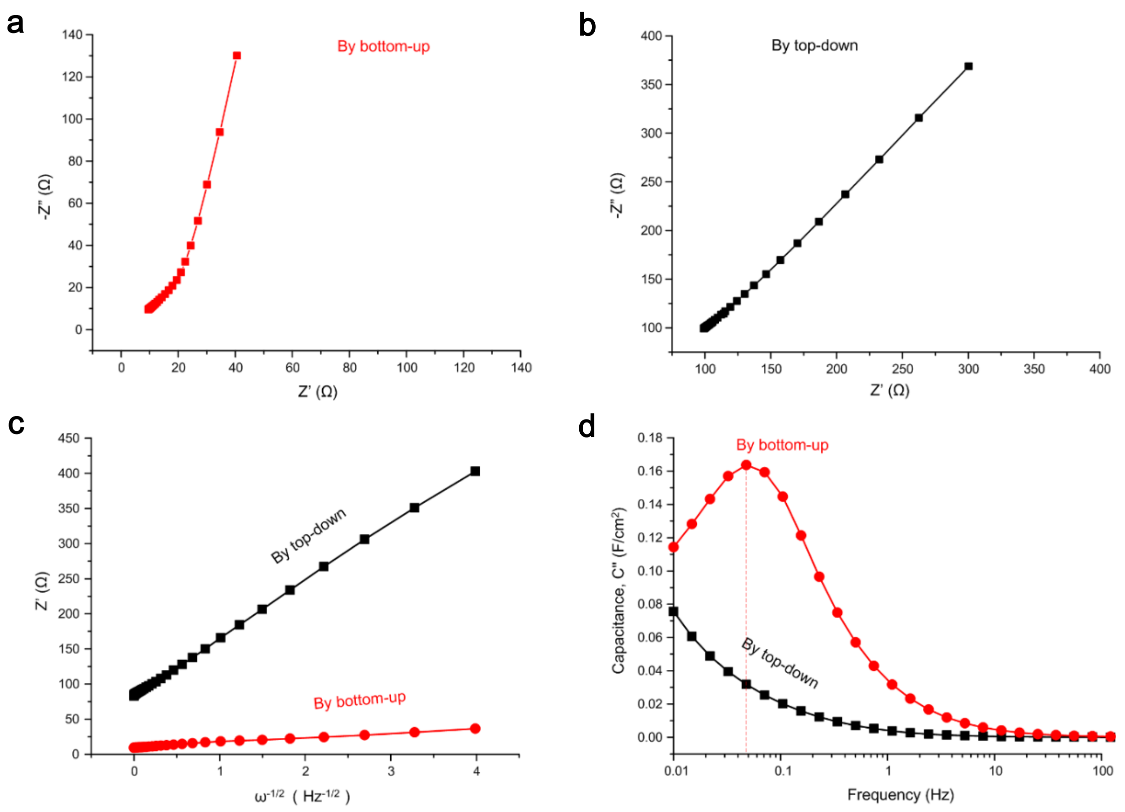

Supplementary Figure 6. EIS results for top-down and bottom-up infilled FSSCs. (a, b) Nyquist plots of the FSSC devices over the frequency range of 1 MHz to 0.01 Hz measured at equilibrium open circuit potential (~0V). (c) Randles plot and (d) imaginary part of capacitance (*C"*) vs frequency (*f*). Dotted line indicates the frequency at the peak. The slope of plot of *Z'* against $\omega^{-1/2}$ (i.e. Randles plot) corresponds to the Warburg coefficient (k_w), which is related to the ion diffusion coefficient (*D*) as follows: $$k_w = \frac{RT}{n^2F^2A\sqrt{2}}\left(\frac{1}{D^{1/2}C^*}\right) \quad (1)$$ where *R* is the gas constant, *T* is the absolute temperature in Kelvin, *n* is the charge transfer number, *A* is the areal of the electrode surface, and *C** is the ionic concentration. The relaxation time for bottom-up FSSC is ~ 25 s, which is smaller than that (> 100 s) for the top-down FSSC. The smaller relaxation time suggest a smaller mass transport distance within the MWCNT electrodes, resulting from that the well-infilled gel has minimized the voids in the pore space of the MWCNT electrodes.

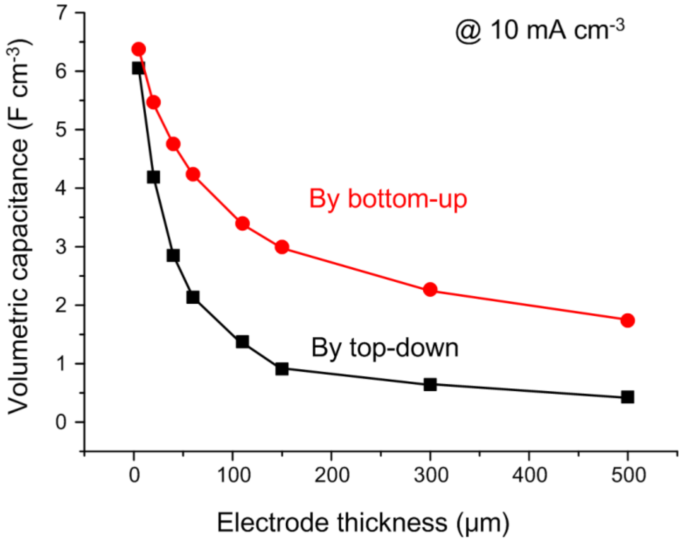

Supplementary Figure 7 | Volumetric capacitance. Thickness dependence of volumetric capacitance of FSSCs formed by the top-down and bottom-up methods.

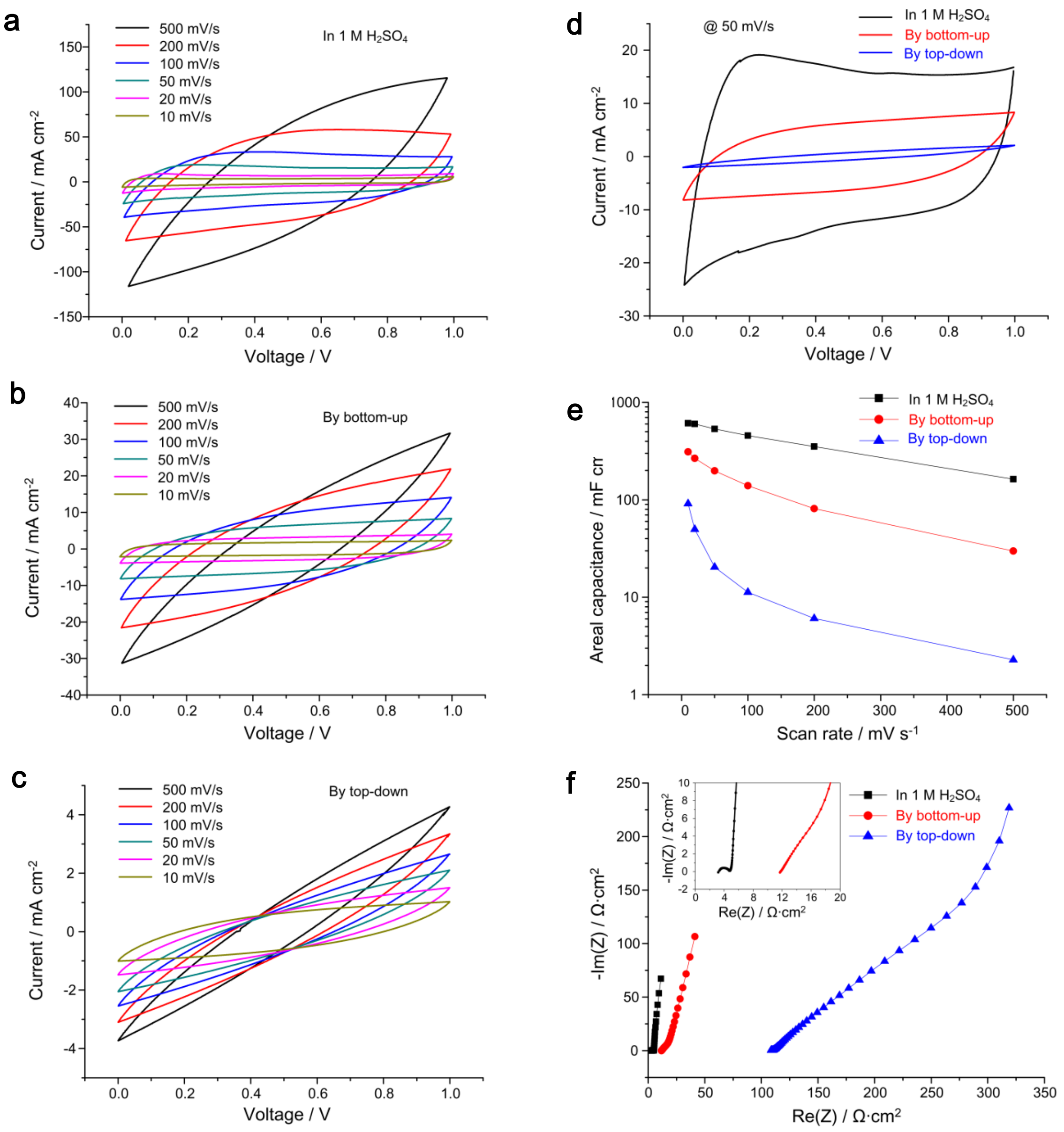

Supplementary Figure 8 | Electrochemical properties over the voltage window of 0 V to 1 V for supercapacitor devices formed using 500 μm thick MWCNT electrodes. (a) CV curves using electrodes infilled with 1 M H_2SO_4, (b) electrodes infilled with the gel electrolyte by the bottom-up method and (c) the top-down method. (d) Typical CV curves of the three different supercapacitors at the same scan rate of 50 mV/s. (e) Areal capacitances of the three different supercapacitors at different current densities. (f) Nyquist plots (from 100 kHz to 10 mHz) of the three different supercapacitors.

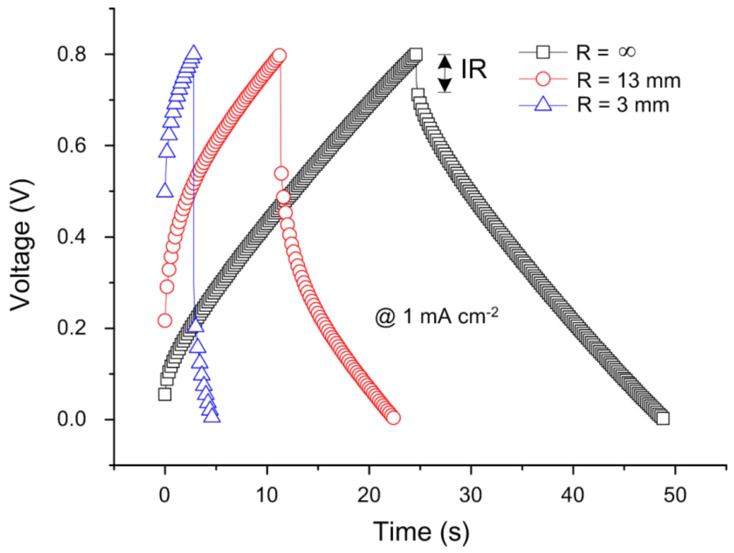

Supplementary Figure 9 | GCD profiles vs. bending radius for a FSSC formed by the top-down infilling method. The ESR values calculated according to the IR drops increased from 28.7 Ω·cm^2 before bending, to 108.8 Ω·cm^2 for a bend radius, R, of 13 mm, and 252.6 Ω·cm^2 for R = 3 mm.

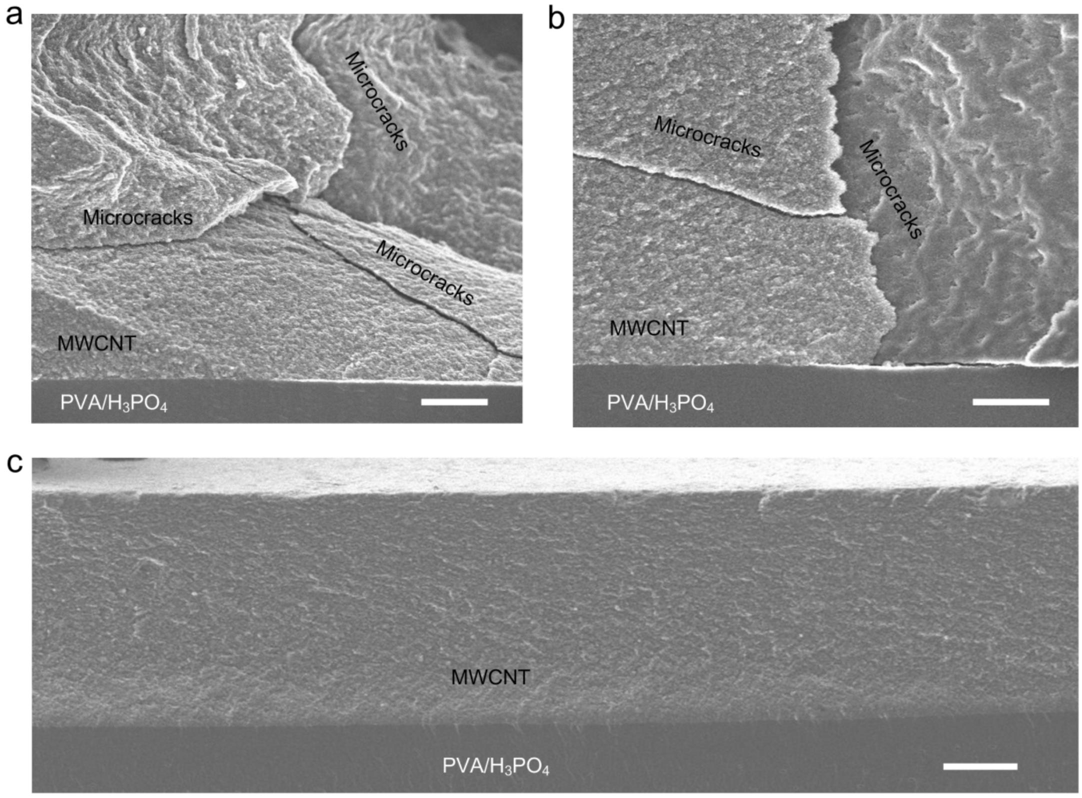

Supplementary Figure 10 | SEM images of different MWCNT electrodes after 5000 bending cycles. Cross-section SEM images of the microcracks (a, b) in the top-down electrode (thickness was 150 μm) of a FSSC device, which has been bent 5000 cycles (bending radius was ~ 5.7 mm). No microcracks were observed in the bottom-up MWCNT electrodes after the same bending cycles (c). Note than the cross section of the electrode was obtained by mechanical fracture in liquid nitrogen. Scale bars are 20μm, 15μm and 50 μm for (a), (b) and (c), respectively.

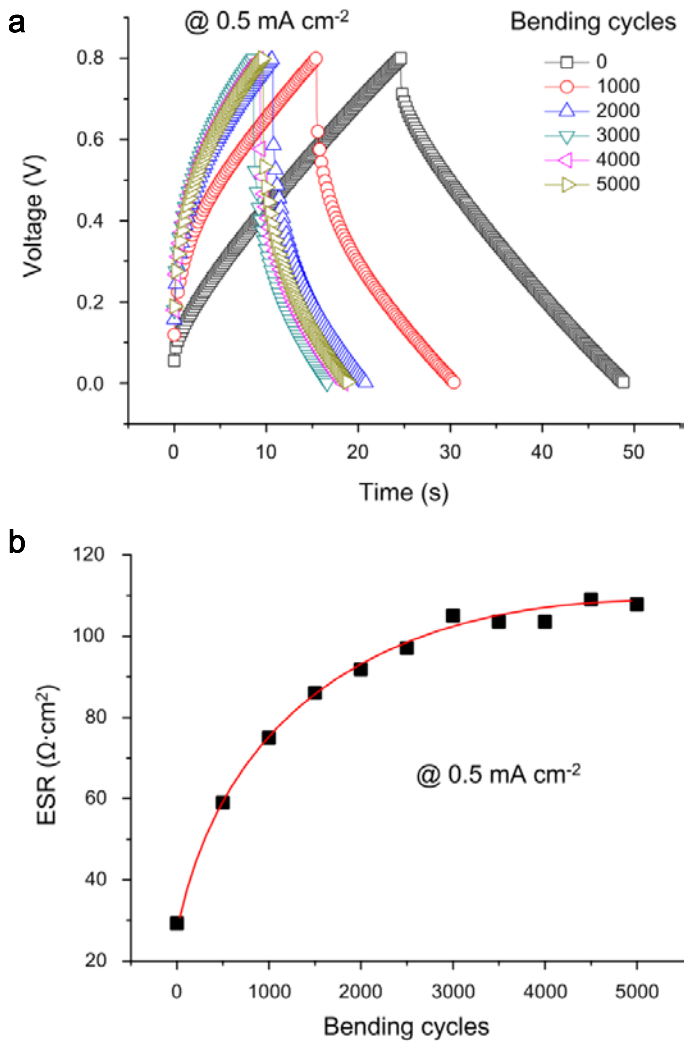

Supplementary Figure 11 | The mechanical durability of a top-down FSSC. (a) GCD profiles vs. the typical number of bending cycles for a FSSC formed by the top-down infilling method. The electrode thickness of the FSSC was ~ 150 μm and the area of the FSSC was 1 cm^2. The GCD profiles of the FSSC were measured after each 500 bend cycles (the bending radius was ~ 5.7 mm). The current density was 0.5 mA cm^{-2}. Note the charging/discharging time significantly decreased and the IR drop increased after 5000 bending cycles. (b) Dependence of ESR on bending cycles. Symbols indicate the calculated ESR values based on the IR drops of the GCD, the curve is a guide to the eye.

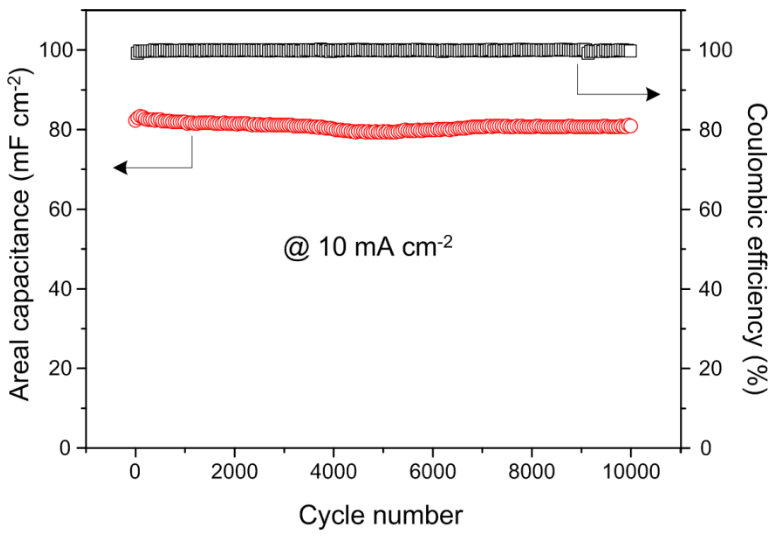

Supplementary Figure 12 | Cycle stability and coulombic efficiency of FSSC formed by the bottom-up method. FSSC was evaluated over 10000 GCD cycles at 10 mA cm^{-2}. Note the thickness of each MWCNT electrode in the FSSC was ~150 μm.

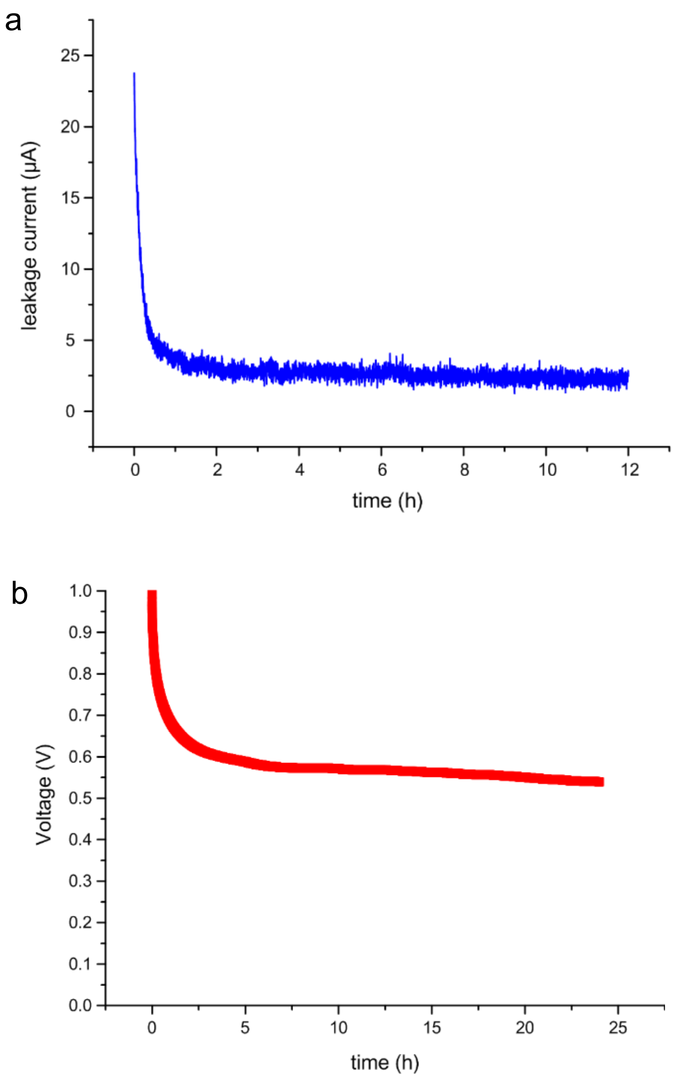

Supplementary Figure 13 | Self-discharge rate of FSSC formed by the bottom-up method. (a) Leakage current measurement of the FSSC. A DC voltage (the voltage at which the supercapacitor is operated, V_{max} = 1 V) was applied across the supercapacitor; the current required to retain that voltage was measured over a period of 12 h. (b) Self-discharge curves of the respective supercapacitors obtained immediately after precharging to V_{max} in the previous test. This involves measuring the open-circuit voltage across the supercapacitor between V_{max} and ½V_{max} vs. time. The electrodes thickness for the used FSSC was ~150 μm.

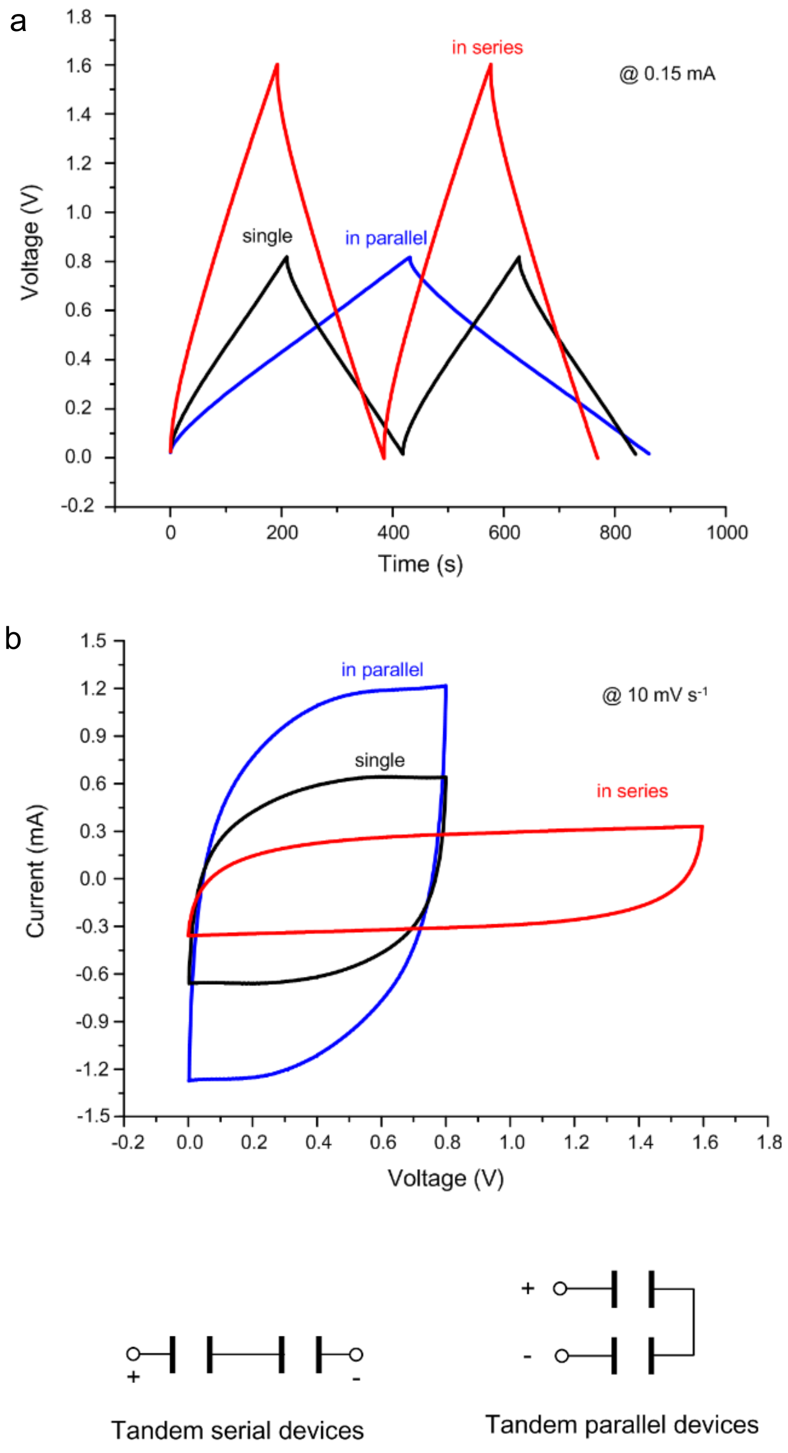

Supplementary Figure 14 | Performance of two tandem FSSCs. The GCD curves at a current of 0.15 mA (a) and CV at a scan rate of 10 mV s^{-1} (b) for tandem FSSC formed by the bottom-up infilling method linked in series and in parallel. The area of each FSSC device was 1 cm^2 and the MWCNT electrodes of each FSSC device was ~ 150 μm.

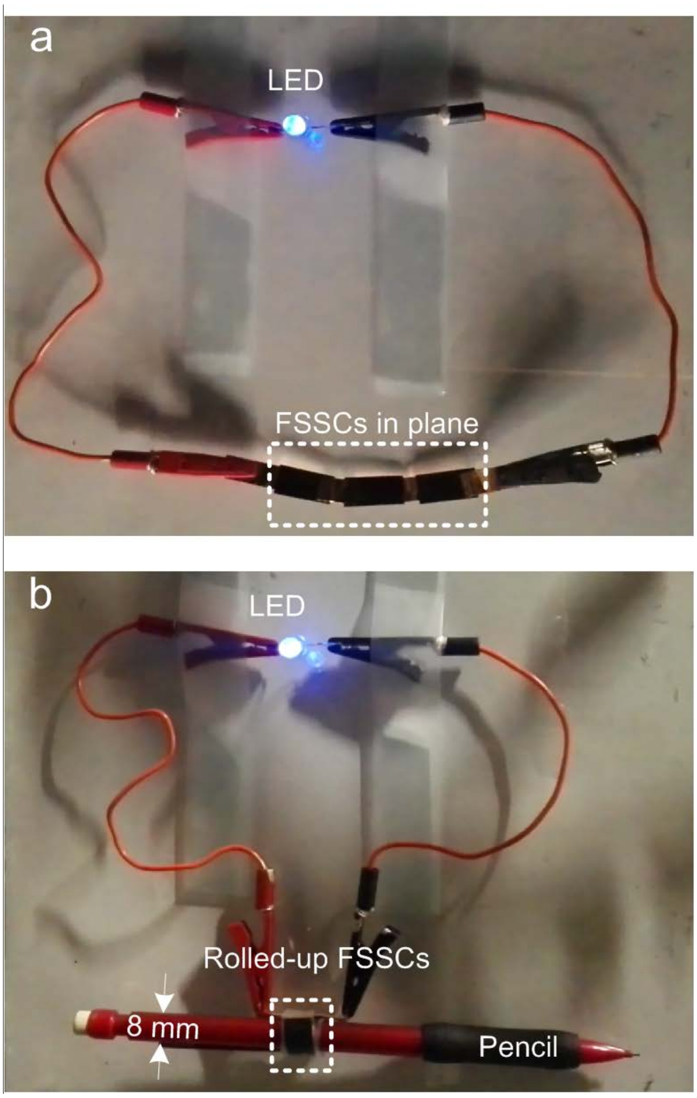

Supplementary Figure 15 | Lighting a blue LED by FSSCs. After charging at a current density of 0.15 mA of 3 V for 23 min, a device of three FSSCs by bottom-up linked in series drove a blue light-emitting-diode (turn-on voltage of ~ 2 V). Note that the rolled-up device around a pencil (b) works as well as when planar (a). The areal of each FSSC was 1 cm^2. The thickness of the each MWCNT electrode was ~ 150 μm.

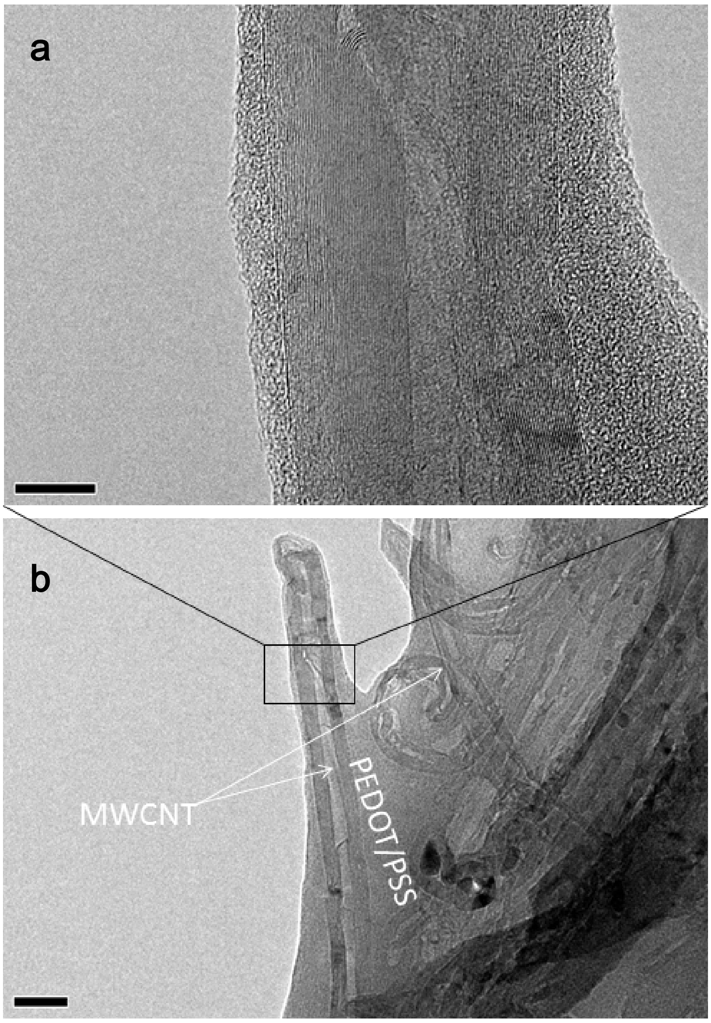

Supplementary Figure 16 | TEM images of the PEDOT/PSS-MWCNT electrode. The lattice of the MWCNT is clear from the high-resolution images while the PEDOT/PSS shows amorphous form (a). The MWCNTs are randomly distributed in the PEDOT/PSS matrix (b). Note that the concentration of PEDOT/PSS in the electrode synthesized as ~70 wt.%. Scale bars: 10 nm for (a) and 50 nm for (b).

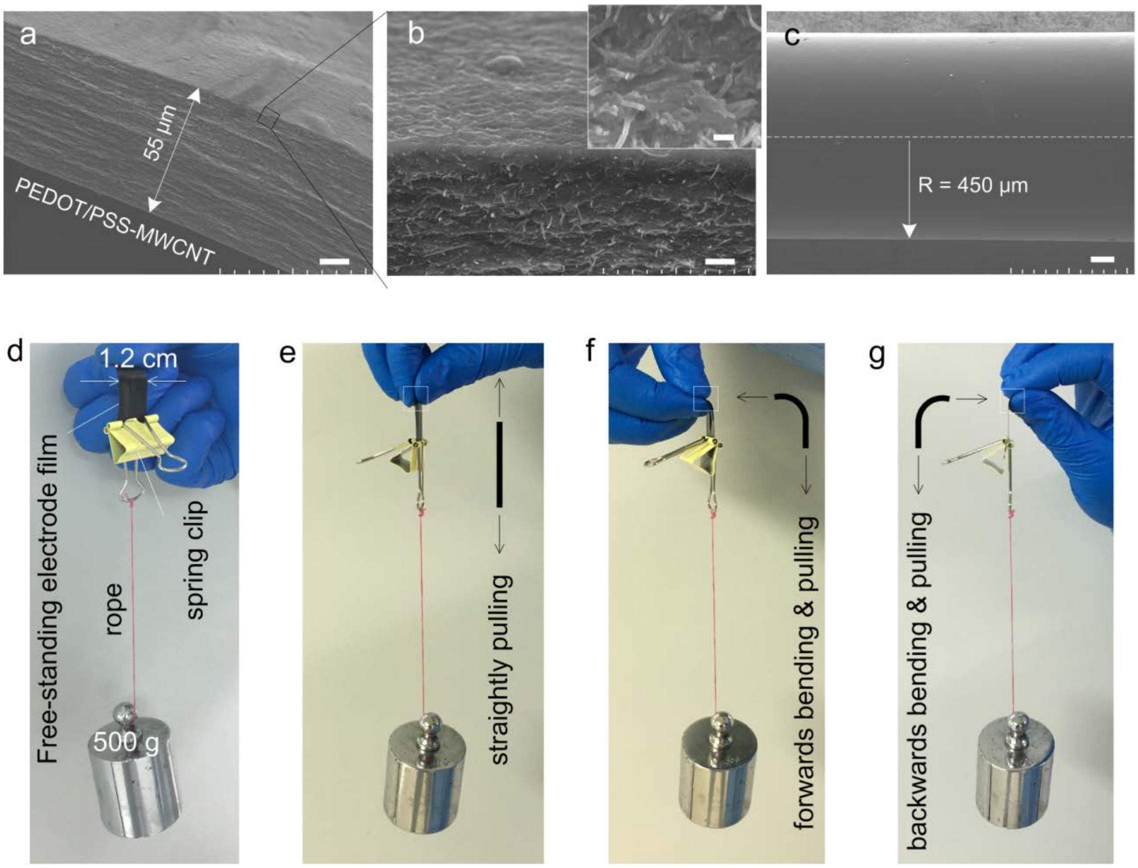

Supplementary Figure 17 | The SEM images and snapshots of PEDOT/PSS-MWCNT films supporting a 500 g mass. SEM images showing the cross section (a, b) and rolled shape (c) of a free-standing PEDOT/PSS-MWCNT electrode. The inset SEM image in (b) shows the uniform distribution of WMCNT and PEDO/PSS. In (c) no microcracks were observed in the electrode film even after bending to a radius of ~ 450 μm. Snapshots of a free-standing PEDOT/PSS-MWCNT electrode thin film (55 μm thick and 12 mm wide), supporting a mass of 500 g (d-g). The free-standing PEDOT/PSS-MWCNT electrode thin film did not fracture or plastically deform under this load (tensile load of 7.5 MPa) when straight or bent forward or backward. Scale bars: 10 μm.

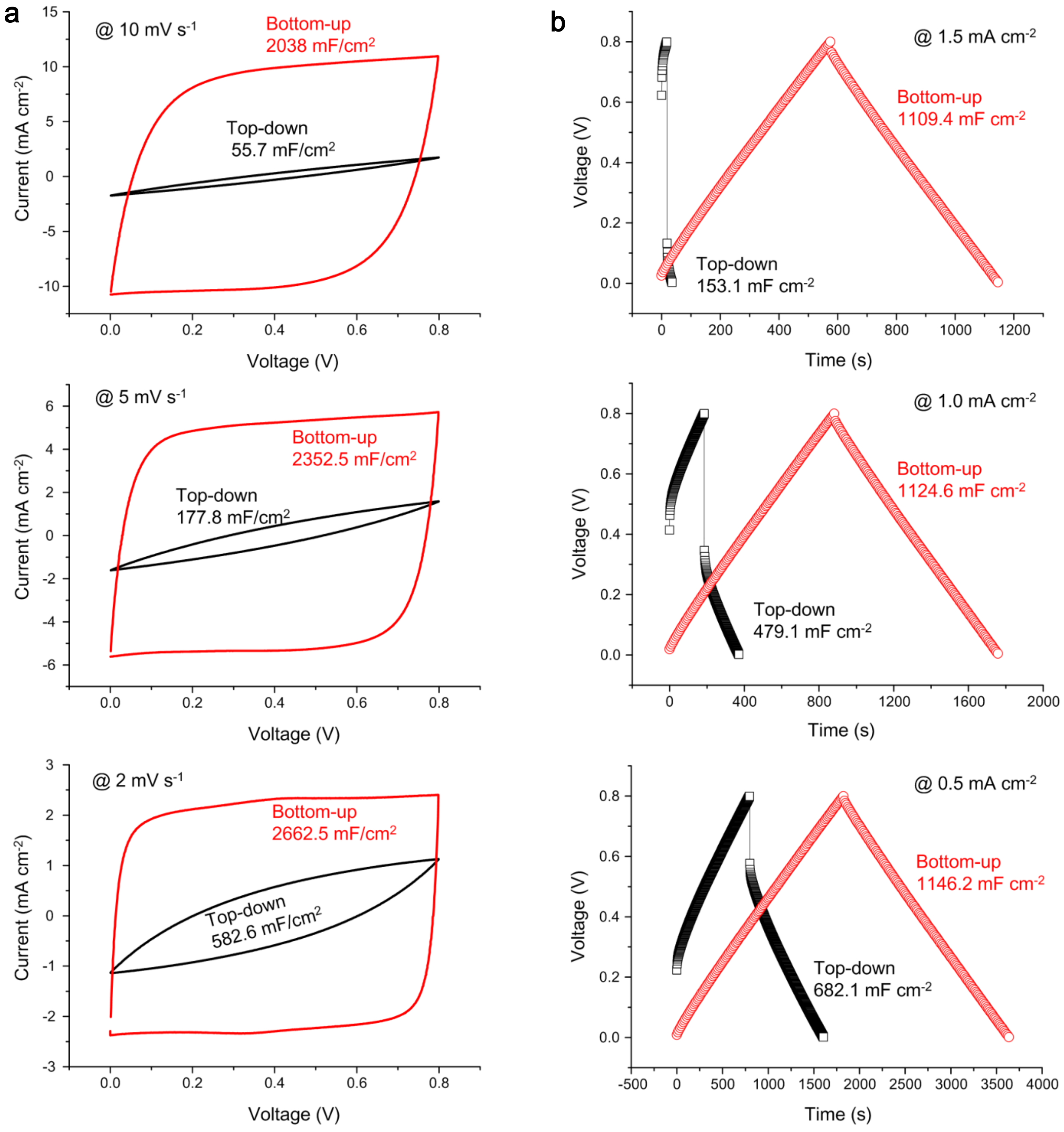

Supplementary Figure 18 | Electrochemical performance of FSSCs with PEDOT/PSS-WMCNT electrodes. CV (a) and GCD (b) profiles at different test conditions for FSSCs with 500-µm-thick PEDOT/PSS-WMCNT electrodes by different designs, as denoted in the figures. Note the different areal capacitances for both top-down and bottom-up infilled FSSCs.

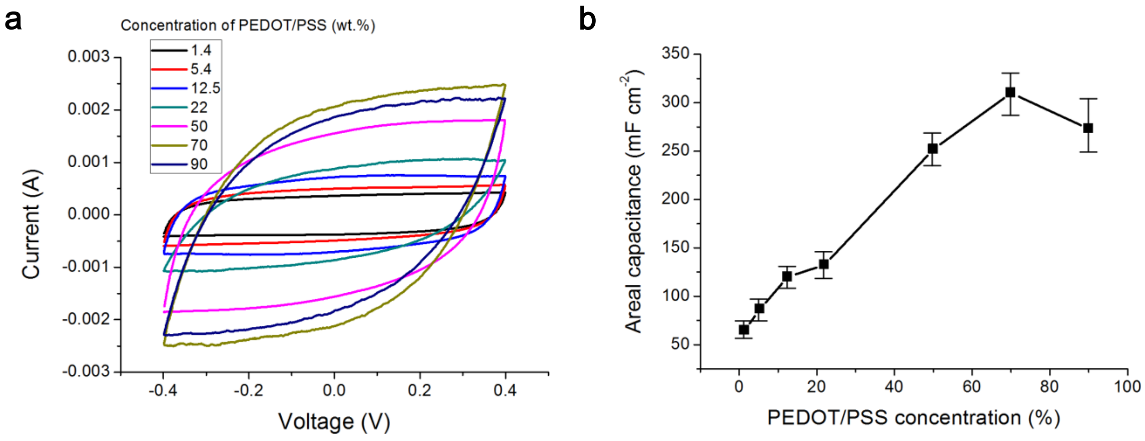

Supplementary Figure 19 | The optimization of PEDOT/PSS concentrations in PEDOT/PSS-MWCNT electrodes. The CV (a) curves of the bottom-up infilled FSSCs with electrodes thickness of generally ~100 μm but different concentrations of PEDOT/PSS. (b) The general dependence of the areal capacitance on the PEDOT/PSS concentrations. The optimized concentration for a maximum areal capacitance is approximately ~ 70 wt.%.

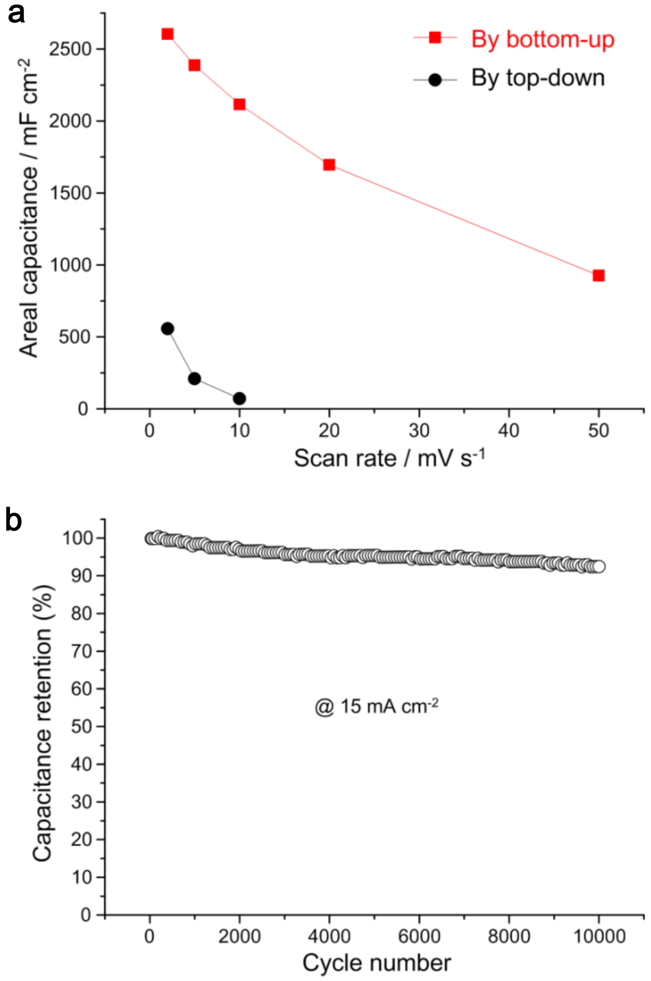

Supplementary Figure 20 | The rate performance and cycle stability of FSSCs with PEDOT/PSS-MWCNT electrodes. (a) Areal capacitances of the top-down and bottom-up infilled FSSCs at different current densities. (b) Cycle stability of FSSC formed by the bottom-up method over 10,000 GCD cycles at 15 mA cm^{-2}. The thickness of each PEDOT/PSS-MWCNT electrode in the FSSCs was ~500 µm.

Supplementary Note 1. The capacitance of each FSSC device was calculated from the GCD profiles at different current densities using the equation:

$C_{device} = i/(-dV/dt)$ (1)

Where i is the current applied (in amps, A), and dV/dt is the slope of the discharge curve (in volts per second, V/s).

The capacitance of FSSC was also calculated from the CV curves at different scan rate using the equation:

$C_{device} = (\int IdV)/(a\Delta V)$ (2)

Where I is the voltammetric current (in amps, A), a is the potential scan rate (in amps, V/s), and V is the potential in one sweep segment.

Specific capacitances were calculated based on the area or the volume of the device stack according to the following formulae:

$C_A = C_{device}/S$ (3)

$C_V = C_{device}/V$ (4)

Where S and V refer to the area (cm^2) and the volume (cm^3) of the device, respectively. The stack capacitances (F cm^{-3}) were calculated based on the total electrode volume excluding the electrolyte.

The areal power (P, in W cm^{-2}) and energy (E, Wh cm^{-2}) were calculated using the following equation:

$E = 0.5C_A (\Delta V)^2 / 3600,\qquad P = E/\Delta t,$ (5)

Where Δt is the discharge time.

The internal resistance was computed from the voltage drop at the beginning of each discharge:

$R_{ESR} = \Delta V_{iR} / 2i$ (6)

Where ΔV_{iR} and i are the voltage drop between the first two points in the voltage drop at the top cutoff and applied current, respectively.
